# Domain-Differentiated Generative AI Competency Self-Efficacy Among University EFL Teachers: Patterns and Predictors

**DOI:** 10.3390/bs16071252

**Published:** 2026-07-22

**Authors:** Aser Altalib

**Affiliations:** Department of English, College of Arts, Jouf University, Sakaka 72341, Saudi Arabia; aaltalib@ju.edu.sa

**Keywords:** generative AI, self-efficacy, AI self-efficacy, domain-specific self-efficacy, teacher AI competence, EFL teachers, professional development, higher education

## Abstract

Self-efficacy, a person’s belief in their capability to carry out a given task, shapes whether teachers attempt, persist with, and transfer new practices. Yet teachers’ perceived capability to use generative AI (GenAI) is often treated as a single general readiness rather than examined across the distinct domains in which it is now used. Guided by Bandura’s self-efficacy theory, this study provides a domain-differentiated account of GenAI competency self-efficacy among university teachers of English as a Foreign Language (EFL) in the Saudi higher-education context. A sample of 232 EFL teachers at Saudi public universities completed a contextually adapted version of the Generative AI Competency Self-Efficacy Scale for Teachers and Researchers (GAICS-TR). Data were analysed using repeated-measures analysis of variance, correlational analyses, and hierarchical multiple regression. Overall self-efficacy was only slightly above the scale midpoint, and the overall score concealed an uneven pattern across domains. Teachers felt most capable in Basic GenAI Understanding and Teaching with GenAI, less so in Professional Engagement, and least confident in Research with GenAI, with the lowest confidence reported for data analysis. Prior AI training and frequency of GenAI use were associated with overall self-efficacy, whereas teaching experience and academic rank were not. The findings suggest that overall measures of GenAI readiness can mask lower-confidence areas within specific domains, especially in research-facing uses such as data analysis. Accordingly, supporting teachers’ development in using GenAI calls for targeted, domain-sensitive professional development that reaches teachers at all career stages rather than broad familiarisation with the tools.

## 1. Introduction

For teachers, the significance of a new technology depends not only on its arrival in their workplace but also on whether they believe they can use it effectively in the tasks their work requires. Generative AI (GenAI) has moved quickly from a novelty to a feature of everyday academic work in higher education, and university teachers are now expected to work with tools such as ChatGPT across teaching, assessment, preparation of materials, feedback, professional learning, and aspects of research (Mohammadi et al., 2026). This expansion has been accompanied by growing interest in how GenAI can support language education and teacher practice (Miao & Cukurova, 2024; Moorhouse et al., 2024). Yet the presence of these tools in academic work does not mean that teachers are prepared to use them confidently, critically, or responsibly.

This is, at its core, a question of perceived capability rather than access or attitude. A teacher may have ready access to GenAI, and may hold broadly favourable views of it, while still feeling uncertain about whether they can use it effectively and responsibly for particular academic tasks. In Bandura’s (1977, 1997) terms, self-efficacy refers to individuals’ beliefs about their capability to organise and carry out courses of action. This matters because perceived capability can shape whether teachers attempt new practices, persist when use becomes demanding, and judge themselves able to transfer a tool from one task to another. In the case of GenAI, such confidence is unlikely to be uniform across academic domains. A teacher might feel assured using GenAI to plan a lesson or draft an assessment, yet considerably less assured using it to design a study, interpret data, or support professional inquiry, because these activities draw on different kinds of knowledge and judgement.

For this reason, domain-differentiated approaches to GenAI competence are important. They make visible the areas in which teachers feel capable and the areas in which confidence remains less secure. This is particularly relevant because much of the emerging literature has examined teachers’ perceptions, acceptance, patterns of use, and AI literacy, rather than the domain structure of their GenAI-related self-efficacy. Recent work has also argued that teacher AI competence should be understood as multidimensional, extending beyond general familiarity with tools to include teaching, assessment, research, professional learning, and ethical use (Xia et al., 2025; Zhou et al., 2026). Without such differentiation, a single overall score may give the impression of general readiness while concealing specific areas in which teachers feel less prepared.

The Saudi university English as a Foreign Language (EFL) context provides a well-suited setting in which to examine this internationally relevant question. Saudi higher education is operating within a wider national environment in which AI has become increasingly prominent in educational and policy discourse (Saudi Press Agency, 2025). At the same time, Saudi EFL research has begun to examine teachers’ perceptions and uses of GenAI tools, showing both recognition of their instructional value and concern about issues such as accuracy, ethical use, and over-reliance (Alkhateeb et al., 2025; Alzubi & Alelaiwi, 2025). What remains less clear is how EFL teachers at Saudi public universities evaluate their own capability across the different domains in which GenAI may now be relevant. Addressing this gap matters because professional development and institutional support are unlikely to be effective if GenAI competence is treated as a single general readiness construct. Against this background, the present study examines GenAI competency self-efficacy among university EFL teachers, drawing on evidence from the Saudi higher-education context and using a domain-differentiated framework, with the aim of informing more precisely targeted support than a single overall measure of readiness would allow.

## 2. Literature Review

### 2.1. GenAI in Language Teaching: Practices and Promise

Few technologies have entered language classrooms as quickly as generative AI. Following ChatGPT’s release, English language teachers began using GenAI tools across the ordinary work of their week: drafting lesson plans, generating and adapting materials, producing reading texts pitched to particular proficiency levels, and giving feedback on student writing and speaking (Moorhouse et al., 2024). The appeal is understandable, given the time pressures under which many teachers work. Studies of pre-service language teachers show similar uses, including lesson planning, development of materials, and refinement of classroom content, often before formal training has caught up with practice (Kerr & Kim, 2025). Among in-service teachers, the pattern is similar: EFL teachers at public higher education institutions in Saudi Arabia have viewed GenAI as useful for material design and planning, teaching methods, assessment, and feedback (Alzubi & Alelaiwi, 2025).

This rapid uptake has often been read as promise: GenAI can support language practice, preparation of materials, feedback, and assessment-related work (Kohnke et al., 2023). Yet cataloguing uses does not show whether teachers are equipped to use the tools well. Generating a text is easy; judging whether its language is accurate, level-appropriate, and pedagogically sound is not, and that judgement rests on the teacher. The question this opens is not simply whether language teachers use GenAI, but how capable they feel across the range of work it now touches.

### 2.2. From Adoption to Competency Self-Efficacy

That teachers are using GenAI tells us less than it first appears. Much of the research prompted by this rapid uptake has been framed through technology acceptance models that treat adoption or the intention to adopt as the outcome to be explained. Hazzan-Bishara et al. (2025), for instance, found that teachers’ exposure to credible AI information shaped their perceptions of GenAI’s usefulness, which in turn predicted their intention to use it. Work of this kind tells us a great deal about what moves teachers towards GenAI, but less about whether they believe they can use it competently. A teacher may regard GenAI as useful, and intend to adopt it, while still feeling unable to design an AI-supported writing task, evaluate the language a model generates, or guide students in using it responsibly. Approval and perceived capability can diverge.

It is the latter that Bandura’s (1977, 1997) concept of self-efficacy brings into focus: a person’s belief in their capacity to organise and carry out the actions a given task requires. Self-efficacy is not a direct measure of skill, but of the conviction that one can deploy relevant capabilities, and that conviction shapes which tasks people attempt, how much effort they sustain, and how they respond to difficulty. Applied to GenAI, it directs attention away from whether teachers approve of these tools and towards whether they feel able to use them across the demands of their work. What remains unclear is how university EFL teachers perceive that capability across the full span of their professional role rather than across classroom tasks alone.

### 2.3. Conceptualising and Measuring Teacher GenAI Competence

If the question is whether teachers feel able to use GenAI, the relevant work must first be specified. For a university teacher it extends well beyond the classroom: alongside teaching, academics design studies, analyse data, write for publication, and are increasingly expected to engage with GenAI in each of these. Capability in one need not imply capability in another, which makes it important to ask how teacher GenAI competence has been conceptualised and measured.

Recent work has produced dedicated instruments for this purpose. The Teacher Artificial Intelligence Competence Self-Efficacy (TAICS) scale (Chiu et al., 2025), for example, captures AI knowledge, pedagogy, assessment, ethics, human-centred education, and professional engagement, and improves on treating AI-related self-efficacy as a single, undifferentiated construct. It sits alongside broader competence frameworks, including the technological pedagogical content knowledge (TPACK) framework (Mishra & Koehler, 2006) and UNESCO’s AI Competency Framework for Teachers (Miao & Cukurova, 2024). These frameworks map relevant domains of competence; self-efficacy asks whether teachers feel able to act within them.

For university teachers, however, scope matters. TAICS was designed for the K–12 context, whereas university academics combine teaching with research, scholarly writing, and professional engagement. The Generative AI Competency Self-Efficacy Scale for Teachers and Researchers (GAICS-TR) was developed to address this broader role (Xia et al., 2025). Validated with 787 academics in China, it spans four domains: basic GenAI understanding, teaching with GenAI, research with GenAI, and professional engagement with GenAI. Its developers note that existing digital and AI competency frameworks often overlook GenAI’s implications for research and assessment and call for cross-cultural and discipline-specific adaptation. The present study responds to that call.

### 2.4. Domain-Specific GenAI Competency Self-Efficacy

Treating GenAI competency self-efficacy as four distinct domains, as GAICS-TR does, rests on a straightforward premise: there is little reason to expect teachers’ confidence to hold steady across every kind of academic work. Using GenAI to generate a classroom activity or draft a quiz draws on familiar pedagogical judgement, whereas using it to design a study, analyse data, or interpret findings draws on research skills that many language teachers exercise less often.

International survey evidence lends some weight to this expectation. In a study of academics across twenty countries, GenAI uptake was found to be uneven across contexts, disciplines, and academic practices, taking in writing, preparation, and research-related work to differing degrees (Mohammadi et al., 2026). That evidence concerns patterns of use rather than perceived capability, and it was gathered from publishing academics across many disciplines rather than from language teachers specifically, so it can do no more than indicate where unevenness might lie. What it cannot establish is whether university EFL teachers feel equally capable across the teaching, research, and professional-engagement domains of their work.

This remains an open question rather than a settled one. Examining the domains separately, rather than collapsing them into a single overall self-efficacy score, is what makes it possible to see whether confidence holds across the professional range or falls away in particular areas.

### 2.5. Predictors of GenAI Competency Self-Efficacy

If self-efficacy varies across domains, it may also vary across teachers. Bandura’s (1977, 1997) account identifies mastery experience as the strongest source of self-efficacy. Applied to GenAI, this points towards AI-specific exposure: training and regular use may build direct experience, whereas seniority carries no such guarantee.

Emerging evidence is broadly consistent with this logic, and it is strongest where it is closest to the present context. In a study of Indonesian EFL teachers, tertiary-level teachers reported the highest self-efficacy, and direct mastery experiences were identified as a key influence on teachers’ AI self-efficacy (Hastomo et al., 2025). Among EFL teachers at public higher education institutions in Saudi Arabia, Alzubi and Alelaiwi (2025) found broadly shared perceptions of GenAI’s usefulness across gender, teaching-experience, and AI-tool mastery groups, while teachers identified material design and planning, teaching methods, and assessment and feedback as key areas of influence. Beyond language education, the same broad picture recurs: frequency of AI use predicted pre-service teachers’ AI self-efficacy while demographic and academic-background variables did not (Aksit et al., 2025), and participation in AI-related courses was positively related to teachers’ AI-TPACK and their perceptions of usefulness (Runge et al., 2025).

Findings are not entirely uniform. Mosia and Nannim (2026), for instance, found that self-efficacy predicted adoption intentions more strongly among untrained than trained teachers, suggesting that exposure and confidence interact in ways still being mapped. Still, much of the evidence comes from studies of pre-service teachers, school teachers, or participants from fields outside language education, leaving open whether AI training and GenAI use outweigh seniority indicators among in-service university EFL teachers. The present study takes up that question. Because the design is cross-sectional and frequency of use is conceptually close to perceived capability, these relationships are treated as associations rather than causal effects.

### 2.6. Saudi University EFL Teachers as an Empirical Context

Saudi Arabia offers a timely setting in which to pursue these questions. Recent research has begun to document how GenAI is entering Saudi university EFL practice. Teachers have drawn on AI tools to prepare for examinations (Alkhateeb et al., 2025), and studies of university instructors have examined their perceptions of AI-generative tools, their readiness to work with ChatGPT, and their ethical awareness in using it (Alsuwat, 2026; Alzubi & Alelaiwi, 2025). This work establishes that GenAI is no longer peripheral to the Saudi EFL context, and that the conversation can now move beyond whether teachers regard these tools favourably. The setting is also one of marked policy momentum, with a national AI curriculum introduced across public education from the 2025–2026 academic year (Saudi Press Agency, 2025). This development provides a context in which university teachers may increasingly be expected to work with GenAI in their own practice.

What this body of work has not yet done is examine how EFL teachers at Saudi public universities perceive their own capability across the full professional range that GenAI now reaches. The issue is not their attitudes towards it, nor their use of it for a particular task, but their self-efficacy across teaching, research, and professional engagement. The present study addresses this gap by applying the four-domain structure of the GAICS-TR to EFL teachers at Saudi public universities, a group for which domain-differentiated evidence remains limited. Its contribution is therefore empirical and contextual: it examines how GenAI competency self-efficacy is distributed across the professional domains represented in an existing higher-education framework and how AI-specific experience and career background are associated with overall self-efficacy in this setting.

### 2.7. The Current Study

Taken together, the strands reviewed above point to a question that existing work has not yet answered. GenAI has become increasingly visible in language teaching, yet research has attended more closely to adoption and approval than to whether teachers believe they can use these tools competently. Where competence has been addressed, it has often been mapped through frameworks specifying what teachers should know and do, or measured through instruments built for school classrooms. Less attention has gone to whether university teachers feel capable across teaching, research, and professional engagement, and Saudi EFL research has focused more on attitudes, readiness, and particular uses than on perceived capability across that professional range.

This study addresses those gaps among a sample of EFL teachers at Saudi public universities, using a contextually adapted GAICS-TR. It examines the level of GenAI competency self-efficacy these teachers report, the domains in which they feel strongest and weakest, and the background and AI-experience factors associated with their overall self-efficacy. Three research questions are addressed:

RQ1. What levels of GenAI competency self-efficacy do EFL teachers at Saudi public universities report?

RQ2. Which domains of GenAI competency self-efficacy are perceived as strongest and weakest among EFL teachers at Saudi public universities?

RQ3. Are prior AI training, frequency of GenAI use, university teaching experience, and academic rank associated with overall GenAI competency self-efficacy?

## 3. Materials and Methods

### 3.1. Research Design

The present study employed a quantitative, cross-sectional survey design, using an online self-report questionnaire administered to EFL teachers at Saudi public universities. The questionnaire comprised a background section and a contextually adapted version of the GAICS-TR (Xia et al., 2025). The design was descriptive, comparative, and correlational: the study examined levels of GenAI competency self-efficacy, compared its four domains, and tested whether selected professional and AI-experience variables were associated with overall self-efficacy.

### 3.2. Setting and Participants

The study was conducted in the Saudi higher-education context, with participants recruited through networks spanning six public universities. The term EFL teachers at Saudi public universities refers here to university-based English-language teaching staff working in Saudi English/EFL programmes across academic ranks, from teaching assistants and language instructors to professors. Their teaching responsibilities may include general language-skills courses and related areas within English departments and programmes.

Participants were recruited through convenience and snowball sampling. The survey link was circulated through departmental and professional networks, with recipients and departmental contacts encouraged to forward it to relevant EFL/English-teaching staff. Because the link was distributed through network forwarding rather than a closed invitation list, the number of individuals who received the survey and the response rate could not be determined. This approach was adopted because access to the target group depended on institutional and professional contacts rather than on an available national sampling frame. The final analytic sample comprised 232 EFL teachers at Saudi public universities. As detailed in Section 3.5, all submitted responses were complete on the GAICS-TR items and the background variables used in the analyses, and no cases were excluded. The sample varied in academic rank, university teaching experience, prior AI training, and GenAI use frequency; the demographic, professional, and AI-related profile is reported in Section 4.

Institutional affiliation was recorded using an optional item. The item was made optional to reduce perceived identifiability among the target population, although the survey collected no names or email addresses. Of the 232 participants, 198 reported an institutional affiliation and 34 did not. All six universities were represented among those who reported. The universities are anonymised as Universities A to F, with reported counts of 55, 39, 36, 27, 22, and 19. Because the affiliation data were optional and incomplete, and because the reported institutional groups were few and unevenly sized, neither multilevel modelling nor cluster-robust standard errors were considered sufficiently reliable for these data. The analyses therefore treat the sample as a single pooled group of EFL teachers at Saudi public universities.

### 3.3. Instruments

Data were collected using an online questionnaire with two parts. The background section collected information on gender, age group, highest qualification, academic rank, university teaching experience, prior AI training, frequency of GenAI use, and the GenAI tools used. Highest qualification and academic rank were recorded through separate questionnaire items; neither variable was derived from or recoded on the basis of the other. Of these, four were treated as the focal variables for the regression analysis: academic rank, university teaching experience, prior AI training, and frequency of GenAI use. The remaining variables were collected to describe the sample. The second part contained the adapted GAICS-TR.

GenAI competency self-efficacy was measured using a contextually adapted version of the Generative Artificial Intelligence Competency Self-Efficacy Scale for Teachers and Researchers (GAICS-TR), adapted from Xia et al. (2025). The original scale comprises 35 items rated from 1 (strongly disagree) to 5 (strongly agree), with higher scores indicating greater self-efficacy. The items cover four domains and nine dimensions: Basic GenAI Understanding (GenAI Knowledge and GenAI Ethics), Teaching with GenAI (Student Interaction, Learning Design, and Assessment), Research with GenAI (Research Design and Data Analysis), and Professional Engagement with GenAI (Professional Development and Staying Current). The scale was developed for higher-education academics and measures perceived capability rather than attitude or acceptance, making it well aligned with the present study’s focus. The item on frequency of GenAI use recorded how often respondents used GenAI, whereas the GAICS-TR elicited self-ratings; respondents who reported never using GenAI could therefore still indicate the extent to which each of its 35 statements applied to them.

Adaptation was confined to contextual wording. Items referring generically to teaching, classroom interaction, or teaching materials were reworded for the Saudi university EFL context; for instance, teaching was specified as English language teaching and students as EFL students. The four domains and item structure were retained unchanged. Three subject-matter experts in applied linguistics, educational technology, and EFL teacher education reviewed the adapted items for clarity, relevance, and contextual fit. The instrument was then piloted with 10 teachers from the target population, who were excluded from the main sample; the pilot examined item clarity, interpretation, contextual relevance, and approximate completion time rather than psychometric properties. No problems requiring revision to the adapted items emerged. Two participants noted that the questionnaire was somewhat long, but the full 35-item set was retained to preserve the source instrument’s coverage and structure. The questionnaire was administered in English because English is the participants’ professional working language and because this preserved equivalence with the source instrument.

### 3.4. Data Collection Procedures

Data were collected through an online survey hosted on Microsoft Forms from 18 January to 4 May 2026, during the second semester of the 2025/2026 academic year. Ethical approval was obtained from Jouf University’s Permanent Ethics Committee before data collection. The opening page contained the information and consent form, which described the study’s aims, the voluntary and anonymous nature of participation, the absence of identifying information, and participants’ right to withdraw before submission. Participants provided electronic informed consent before proceeding. No personally identifying information, such as names or email addresses, was recorded, no incentive was offered, and responses were stored securely and used only for this research.

### 3.5. Data Analysis

The main analyses were conducted using IBM SPSS Statistics (Version 29), and the confirmatory factor analyses were conducted in R (Version 4.6.1; R Core Team, 2026) using the lavaan package (Version 0.6-20; Rosseel, 2012; Rosseel et al., 2025). The exported dataset contained 232 submitted responses. Because the GAICS-TR items and the background questions used in the analyses were set as required in Microsoft Forms, all submitted cases had complete data for those variables. The data were inspected for completeness and uniform response patterns across the 35 GAICS-TR items. Exact straight-lining was operationalised as zero within-case variance, that is, the same response to every item. No case met this criterion, and no cases were excluded; the final analytic sample therefore comprised all 232 submitted responses. Dimension, domain, and overall GAICS-TR scores were computed as mean scores from their assigned items; no items were reverse-coded, and no imputation, prorating, or minimum-item threshold was required. Internal consistency was assessed using Cronbach’s alpha for the total scale and the four domains.

Because the GAICS-TR was contextually adapted for EFL teachers at Saudi public universities, its measurement structure was examined in the present sample. Item-level confirmatory factor analysis (CFA) was conducted for the 35 adapted items using the published four-domain structure. Given the ordinal response format, the four-domain, one-factor, and nine-factor item-level models were first estimated using weighted least squares mean- and variance-adjusted (WLSMV), with items treated as ordered categorical indicators. The four-domain and one-factor item-level models were also examined using robust maximum likelihood (MLR) as estimator-sensitivity checks. The substantive analyses used pre-specified composite scores at the dimension, domain, and overall levels. A supplementary dimension-level CFA was therefore conducted, using the nine dimension scores as indicators of the four domains; a one-factor dimension-level model was also estimated for comparison. Factor loadings, factor correlations, average variance extracted (AVE), and evidence concerning discriminant separation were inspected. The measurement-check results are reported in Section 4.

Effect sizes were reported alongside significance tests and interpreted using Cohen’s (1988) conventions, with partial eta-squared values of 0.01, 0.06, and 0.14 taken as small, medium, and large, and Pearson correlations of 0.10, 0.30, and 0.50 taken as small, medium, and large.

RQ1 was addressed using descriptive statistics, including means, standard deviations, confidence intervals, medians, trimmed means, and distributional indices where relevant; means were interpreted in relation to the 1–5 response scale and its midpoint.

RQ2 was addressed using a one-way repeated-measures analysis of variance, with domain as the within-subjects factor. Where Mauchly’s test indicated a violation of sphericity, the Greenhouse–Geisser correction was applied, and Bonferroni-adjusted comparisons examined pairwise domain differences. A Friedman test was also conducted as a nonparametric robustness check.

RQ3 was addressed through bivariate correlations and hierarchical multiple regression, with overall GenAI competency self-efficacy as the outcome. Pearson and Spearman correlations were inspected for the focal predictors. In the regression, Step 1 entered university teaching experience and academic rank; Step 2 added prior AI training and frequency of GenAI use to examine AI-specific experience beyond career background. Highest qualification was not entered because it was analytically redundant with academic rank in this sample, and the remaining background and tool-use variables were descriptive rather than focal predictors. Regression diagnostics checked multicollinearity using tolerance and variance inflation factor (VIF) values, and influential cases using Cook’s distance. Given the cross-sectional design, regression results were interpreted as associations rather than causal effects.

The four focal predictors were entered into the main hierarchical regression as ordered numeric codes. Academic rank and university teaching experience were coded from 1 to 5 in ascending category order, AI training from 0 to 3, and GenAI use frequency from 0 to 4, following the category order shown in Table 1 and Table 2. The ordered specification was used as a parsimonious primary model, estimating a single trend across the categories rather than a separate coefficient for each. This treatment assumes approximately equal spacing between adjacent categories and an approximately linear relationship with overall GenAI competency self-efficacy. To assess whether the findings depended on this assumption, the final Step 2 model was re-estimated with the four predictors entered as dummy-coded contrasts. The reference categories were no AI training, never using GenAI, five years or less of university teaching experience, and Lecturer. For teaching experience, the two lowest categories were combined because the less-than-one-year category contained only one case. The AI-training variable was interpreted as an ordered exposure proxy because its categories combine type and amount of training. The regression assumptions were examined through diagnostics for linearity, normality of residuals, homoscedasticity, and independence of errors, in addition to the multicollinearity and influence checks already described. The stability of the estimates was further examined using 95% bias-corrected and accelerated (BCa) bootstrap confidence intervals based on 5000 resamples and robust standard errors from an equivalent generalised linear model with a normal distribution, identity link, and robust covariance estimation. As a limited common-method diagnostic, Harman’s single-factor analysis was conducted on the 35 GAICS-TR items. Sensitivity analyses re-estimated the repeated-measures domain comparisons and the regression after excluding the 36 respondents who reported never using GenAI, and re-estimated the regression with gender and collapsed age groups added as controls. The results of these checks are reported in Section 4.

## 4. Results

### 4.1. Preliminary Analyses and Sample Characteristics

Table 1 presents the demographic and professional profile of the sample. The 232 participants were relatively balanced by gender, comprising 123 males (53.0%) and 109 females (47.0%). Most were aged between 30 and 49 years, with 108 (46.6%) in the 30–39 band and 84 (36.2%) in the 40–49 band. The sample was academically experienced: 100 respondents (43.1%) held a master’s degree and 96 (41.4%) held a PhD or doctorate. Lecturers formed the largest rank group (*n* = 100, 43.1%), followed by assistant professors (*n* = 47, 20.3%), teaching assistants or language instructors (*n* = 36, 15.5%), associate professors (*n* = 27, 11.6%), and professors (*n* = 22, 9.5%). The joint distribution of qualification and academic rank showed complete alignment in this sample: all 36 respondents with a bachelor’s degree were teaching assistants or language instructors, all 100 respondents with a master’s degree were lecturers, and all 96 respondents with a PhD or doctorate were assistant professors (*n* = 47), associate professors (*n* = 27), or professors (*n* = 22). No other qualification–rank combinations occurred. This alignment is consistent with the qualification-linked appointment structure of Saudi public universities: a bachelor’s degree corresponds to teaching assistant or language instructor positions, a master’s degree to lecturer positions, and a doctorate to the professorial ranks, with progression from assistant professor to associate professor and professor determined by experience and promotion requirements. As noted in Section 3.3, the variables were recorded independently; the observed pattern is therefore not the result of deriving or recoding one variable from the other. Most respondents reported substantial university-level teaching experience, with 86 (37.1%) reporting 6–10 years, 77 (33.2%) reporting 11–15 years, and 56 (24.1%) reporting more than 15 years.

Table 2 summarises participants’ AI-related background. A substantial proportion of the sample had received no training in using GenAI for teaching or academic work (*n* = 100, 43.1%). A further 65 (28.0%) reported informal or self-directed learning only, while 39 (16.8%) reported one formal training session or workshop and 28 (12.1%) reported multiple formal sessions or a structured course. In other words, although more than half of the respondents had some exposure to training or self-directed learning, only 67 (28.9%) reported any formal training. Frequency of GenAI use also varied: 36 respondents (15.5%) reported never using GenAI, 46 (19.8%) reported rare use, 56 (24.1%) occasional use, 56 (24.1%) frequent use, and 38 (16.4%) daily or almost daily use. Together, frequent and daily users accounted for 94 respondents (40.5%). In terms of tools, ChatGPT was by far the most commonly selected (*n* = 155, 66.8%), followed by Google Gemini (*n* = 54, 23.3%), Grammarly or AI writing assistants (*n* = 44, 19.0%), Claude (*n* = 35, 15.1%), and Microsoft Copilot (*n* = 11, 4.7%); no respondent selected DeepSeek. Tool-use variables were reported descriptively only and were not used as predictors.

Internal consistency was examined before the substantive analyses. Cronbach’s alpha was high for the full 35-item scale (*α* = 0.945) and good for Basic GenAI Understanding (*α* = 0.863), Teaching with GenAI (*α* = 0.874), and Professional Engagement with GenAI (*α* = 0.854). For Research with GenAI, it was acceptable (*α* = 0.778), the lowest of the four domains. Taken together, these coefficients indicate adequate internal consistency for the total and domain composite scores (Table 3), but they do not establish that the four domains are empirically distinct in this sample; the measurement structure is examined separately in Section 4.2.

### 4.2. Measurement Checks for the Adapted GAICS-TR

Before the substantive analyses, the measurement structure of the adapted GAICS-TR was examined through confirmatory factor analysis, with the results summarised in Table 4. At the item level, the four-domain model estimated with WLSMV did not fit the data acceptably. A one-factor item-level model showed poorer fit. Re-estimating both the four-domain and one-factor item-level models with MLR led to the same substantive conclusion: neither fitted acceptably, indicating that the poor item-level fit was not specific to WLSMV estimation. A nine-factor item-level model was also estimated, but the solution was inadmissible because the latent covariance matrix was not positive definite and several latent correlations exceeded one in absolute value; it was therefore not interpreted. Taken together, the item-level analyses did not provide acceptable evidence for a clean item-level latent structure in this sample.

Because the substantive analyses used pre-specified composite scores rather than item-level latent factor scores, a supplementary dimension-level model was examined, with the nine dimension scores specified as indicators of the four domains. This dimension-level four-domain model showed acceptable fit overall (Table 4) and fitted substantially better than a one-factor dimension-level model (Δ*χ*^2^(6) = 173.47, *p* < 0.001). Standardised loadings ranged from 0.731 to 0.967, and average variance extracted (AVE) values ranged from 0.725 to 0.856. However, Basic GenAI Understanding and Teaching with GenAI were very highly correlated (*r* = 0.959). The squared correlation (0.919) exceeded the AVE for both domains, indicating substantial overlap and limited discriminant separation between them. These two domains are therefore interpreted as overlapping rather than sharply distinct in the domain-level results that follow. These findings are treated as supportive evidence for the four-domain scoring structure used in the substantive analyses, not as validation of the adapted instrument.

### 4.3. Levels of GenAI Competency Self-Efficacy (RQ1)

The first research question asked what levels of GenAI competency self-efficacy the surveyed teachers reported. The overall GAICS-TR mean was 3.23 (*SD* = 0.64) on the five-point scale, with a 95% confidence interval of [3.15, 3.32]. This places overall self-efficacy slightly above the neutral midpoint of the scale, but not at a level that would describe the sample as highly confident. The distribution was approximately symmetric: the median was 3.23, the 5% trimmed mean was 3.24, and skewness (−0.264) and kurtosis (0.545) were both within conventional bounds, indicating that the mean was not distorted by extreme values.

At the domain level, the pattern was uneven (Table 5). Basic GenAI Understanding and Teaching with GenAI had the two highest means (*M* = 3.42, *SD* = 0.80; *M* = 3.37, *SD* = 0.77, respectively). Professional Engagement with GenAI was somewhat lower but still above the midpoint (*M* = 3.27, *SD* = 0.73), whereas Research with GenAI was the only domain to fall below the midpoint (*M* = 2.84, *SD* = 0.62), marking it as the clear area of relative weakness.

The nine-dimension descriptives in Table 6 show where this domain pattern originates. The highest dimension means were for GenAI Ethics (*M* = 3.52, *SD* = 0.91), GenAI for Assessment (*M* = 3.43, *SD* = 0.79), and GenAI for Learning Design (*M* = 3.41, *SD* = 0.81), all sitting within the stronger Basic Understanding and Teaching domains. The two research-facing dimensions were the lowest by a clear margin: GenAI for Research Design (*M* = 2.94, *SD* = 0.66) and GenAI for Data Analysis (*M* = 2.73, *SD* = 0.67). In other words, the weakness of the Research with GenAI domain was reflected in both of its constituent dimensions, with data-analysis-related confidence the lowest of all nine. At the item level, the highest means clustered around security, content generation, and assessment-related uses of GenAI, whereas the lowest means were concentrated in data-analysis-related items, reinforcing the weaker Research with GenAI profile shown in the dimension-level results.

Taken together, the answer to RQ1 is that the surveyed teachers reported overall GenAI competency self-efficacy only slightly above the scale midpoint. Their confidence was unevenly distributed across professional domains: it was strongest in basic understanding and teaching-related applications, intermediate in professional engagement, and weakest in research-related uses, particularly data analysis.

### 4.4. Domain-Level Differences in GenAI Competency Self-Efficacy (RQ2)

The second research question asked which domains of GenAI competency self-efficacy were perceived as strongest and weakest. The domain descriptives reported in Table 5 indicated an uneven profile and were therefore followed by a one-way repeated-measures analysis of variance, with the four GAICS-TR domains as the within-subjects factor.

Mauchly’s test indicated that the assumption of sphericity was violated (*W* = 0.645, *χ*^2^(5) = 100.71, *p* < 0.001), so the Greenhouse–Geisser correction was applied. The analysis revealed a statistically significant difference among the four domains (*F*(2.37, 548.33) = 91.40, *p* < 0.001, *η*p^2^ = 0.283), a large effect. The four domains were therefore not rated equally.

Bonferroni-adjusted pairwise comparisons clarified the pattern (Table 7). Basic GenAI Understanding and Teaching with GenAI did not differ significantly (mean difference = 0.04, *p* = 0.705), indicating that they formed a statistically comparable strongest group. Both were significantly higher than Professional Engagement with GenAI (Basic Understanding, mean difference = 0.15, *p* = 0.001; Teaching, mean difference = 0.11, *p* = 0.010) and higher still than Research with GenAI (Basic Understanding, mean difference = 0.58, *p* < 0.001; Teaching, mean difference = 0.54, *p* < 0.001). Professional Engagement, in turn, was significantly higher than Research with GenAI (mean difference = 0.43, *p* < 0.001). Research with GenAI was thus significantly lower than all three other domains.

A Friedman test, conducted as a nonparametric robustness check, confirmed the same pattern (*χ*^2^(3, *N* = 232) = 167.52, *p* < 0.001). The mean ranks followed the identical order: Basic GenAI Understanding was highest (3.03), followed by Teaching with GenAI (2.80), Professional Engagement (2.51), and Research with GenAI (1.66).

Excluding the 36 respondents who reported never using GenAI did not alter the domain hierarchy. Among the remaining 196 respondents, a Friedman test confirmed an overall difference across the four domains (*χ*^2^(3, *N* = 196) = 165.91, *p* < 0.001). The Bonferroni-adjusted comparisons likewise showed that Basic GenAI Understanding and Teaching with GenAI remained statistically comparable and jointly highest, Professional Engagement remained intermediate, and Research with GenAI remained significantly lower than all three other domains.

In sum, the answer to RQ2 is that the surveyed teachers reported their strongest GenAI competency self-efficacy in basic understanding and teaching-related uses, which were statistically comparable; an intermediate level in professional engagement; and their weakest self-efficacy in research-related uses, which fell significantly below every other domain.

### 4.5. Predictors of Overall GenAI Competency Self-Efficacy (RQ3)

The third research question asked whether prior AI training, frequency of GenAI use, university teaching experience, and academic rank were associated with overall GenAI competency self-efficacy. Bivariate correlations were inspected first, using both Pearson and Spearman coefficients given the ordinal nature of the predictors (Table 8). Overall GAICS-TR scores were strongly and positively correlated with frequency of GenAI use (*r* = 0.730, *p* < 0.001; *ρ* = 0.756, *p* < 0.001) and with prior AI training (*r* = 0.671, *p* < 0.001; *ρ* = 0.663, *p* < 0.001). Teaching experience and academic rank, by contrast, were not significantly correlated with overall self-efficacy using either Pearson coefficients (experience *r* = −0.077, *p* = 0.241; rank *r* = −0.110, *p* = 0.096) or Spearman coefficients (experience *ρ* = −0.058, *p* = 0.377; rank *ρ* = −0.082, *p* = 0.214).

As reported in Section 4.1, highest qualification and academic rank were completely aligned in this sample. Qualification was therefore not entered into the regression because it was analytically redundant with academic rank.

A hierarchical multiple regression was then estimated with overall GenAI competency self-efficacy as the outcome (Table 9). Step 1, which entered university teaching experience and academic rank, was not statistically significant (*R*^2^ = 0.013, adjusted *R*^2^ = 0.004, *F*(2, 229) = 1.49, *p* = 0.227). The addition of prior AI training and frequency of GenAI use in Step 2 substantially improved the model, which became statistically significant (*R*^2^ = 0.665, adjusted *R*^2^ = 0.659, *F*(4, 227) = 112.80, *p* < 0.001). The two AI-specific variables accounted for a large additional share of variance beyond career background (Δ*R*^2^ = 0.652, *F* change (2, 227) = 221.23, *p* < 0.001).

In the final model, frequency of GenAI use was the strongest predictor (*B* = 0.261, *β* = 0.533, *t* = 12.023, *p* < 0.001), followed by prior AI training (*B* = 0.252, *β* = 0.410, *t* = 9.177, *p* < 0.001). University teaching experience was not a statistically significant predictor in either model step, although its coefficient became more negative after the AI-specific variables were added, changing from *B* = −0.024 in Step 1 to *B* = −0.058 in Step 2 (*β* = −0.081, *p* = 0.065). Academic rank was likewise not a statistically significant predictor in the final model (*B* = 0.028, *β* = 0.051, *p* = 0.248). Collinearity was low (VIF values from 1.284 to 1.356) and influence diagnostics were acceptable (maximum Cook’s distance = 0.070), indicating no serious threat to the model.

Several checks supported the stability of the regression findings. Re-estimating the final Step 2 model with the four predictors entered as dummy-coded contrasts produced the same substantive pattern. The dummy-coded model was statistically significant (*R*^2^ = 0.682, adjusted *R*^2^ = 0.662, *F*(14, 217) = 33.251, *p* < 0.001). Relative to no AI training, the estimated coefficients were *B* = 0.162 (*p* = 0.024) for informal or self-directed learning only, *B* = 0.416 (*p* < 0.001) for one formal training session or workshop, and *B* = 0.740 (*p* < 0.001) for multiple formal training sessions or a structured course. Relative to never using GenAI, they were *B* = 0.176 (*p* = 0.043) for rare use, *B* = 0.459 (*p* < 0.001) for occasional use, *B* = 0.842 (*p* < 0.001) for frequent use, and *B* = 1.018 (*p* < 0.001) for daily or almost daily use. None of the teaching-experience or academic-rank contrasts was statistically significant. The main result therefore did not depend on treating the ordinal predictors as equally spaced.

Diagnostics showed no serious multicollinearity or influence problem. Inspection of the standardised residual-versus-predicted plot did not indicate a marked curvilinear pattern. The Durbin–Watson statistic was 1.495, although its interpretation is limited for unordered cross-sectional data. The standardised residuals showed mild departures from normality (skewness = −0.589, kurtosis = 1.151), and an auxiliary regression of the squared residuals on the four predictors indicated unequal variance (*R*^2^ = 0.105, *F*(4, 227) = 6.640, *p* < 0.001). Given the departures from residual normality and homoscedasticity, BCa bootstrap confidence intervals and robust standard errors were examined. Under both approaches, only AI training and GenAI use frequency remained statistically significant. For prior AI training, *B* = 0.252, with a bootstrap standard error of 0.026 and a 95% BCa confidence interval of [0.200, 0.301]; the corresponding robust standard error was 0.026. For GenAI use frequency, *B* = 0.261, with a bootstrap standard error of 0.023 and a 95% BCa confidence interval of [0.217, 0.308]; the corresponding robust standard error was 0.023. Both coefficients remained significant at *p* < 0.001 under the robust analysis.

In Harman’s single-factor diagnostic, the first unrotated principal component accounted for 35.46% of the variance. This did not suggest a single dominant factor, although the diagnostic cannot rule out common-method effects. In a secondary sensitivity analysis, excluding the 36 respondents who reported never using GenAI and re-estimating the model with the remaining 196 respondents preserved the same predictor pattern (*R*^2^ = 0.694). Adding gender and collapsed age groups explained little additional variance (Δ*R*^2^ = 0.008, *p* = 0.163), and did not alter the pattern for the four focal predictors. The female-versus-male coefficient was small and negative (*B* = −0.116, *β* = −0.090, *p* = 0.025), whereas the age-group contrasts were not statistically significant.

The answer to RQ3, then, is that overall GenAI competency self-efficacy was strongly associated with AI-specific engagement rather than with general academic seniority: prior AI training and frequency of GenAI use were both strong positive predictors, while teaching experience and academic rank were not. The size of the explained variance should be read with caution, however, because the design is cross-sectional and frequency of GenAI use and competency self-efficacy are conceptually proximal; the model therefore indicates a strong association between self-reported use and self-reported capability rather than evidence that more frequent use produces higher self-efficacy.

## 5. Discussion

### 5.1. Overview of Results

This study examined GenAI competency self-efficacy among EFL teachers at Saudi public universities, the domains in which it was stronger or weaker, and the background and AI-experience factors associated with it. The findings indicate that an overall level only slightly above the scale midpoint coexisted with variation across professional domains, while AI-specific experience was more closely associated with self-efficacy than general academic seniority. The sections that follow consider these patterns in relation to each research question and the wider literature.

### 5.2. Levels of GenAI Competency Self-Efficacy

The first research question concerned the levels of GenAI competency self-efficacy the surveyed teachers reported. The overall mean of 3.23 lay just above the scale midpoint. This could indicate an emerging rather than a well-established sense of capability: the surveyed teachers, on average, expressed neither marked uncertainty nor strong confidence about working with GenAI. This level is consistent with a population still in the early stages of integrating GenAI into academic work, where confidence is forming but uneven. This also fits Bandura’s (1977, 1997) view of self-efficacy as built incrementally through mastery experiences. Hastomo et al. (2025) offer a useful comparative point, showing that AI-tool self-efficacy among Indonesian EFL teachers was strongest at the tertiary level; read alongside the present findings, this suggests that university teachers may be well placed to develop GenAI self-efficacy without necessarily reporting uniformly high confidence.

Within this overall picture, the dimensional pattern is informative. Confidence was highest for the ethics-related dimension (*M* = 3.52), ahead of assessment and learning design, and lowest for the two research-facing dimensions. The relative prominence of ethics-related confidence could reflect the salience of ethical and integrity questions in institutional and public discussion of GenAI. This reading aligns with Kohnke et al. (2025), who found that pre-service teachers foregrounded ethical understanding alongside technical skill as central to using GenAI in education, and with Alzubi and Alelaiwi (2025), whose participants reported broadly favourable views of GenAI alongside caution about its ethical use and the risk of over-reliance. It is important to be precise about what this dimension captures, however. Because the instrument measures self-efficacy, the finding speaks to teachers’ confidence in handling ethics-related aspects of GenAI use, not to their actual ethical expertise; a teacher may feel capable of navigating integrity concerns without that perception having been tested against practice. Read at this level, the result suggests that ethical confidence is comparatively well developed in this sample, even as research-related confidence lags behind, a divergence that becomes clearer in the domain-level comparison discussed in Section 5.3.

### 5.3. Domain-Level Differences in GenAI Competency Self-Efficacy

The second research question concerned which domains of GenAI competency self-efficacy were perceived as strongest and weakest. A clear hierarchy emerged. Basic GenAI Understanding and Teaching with GenAI formed a statistically comparable strongest pair, Professional Engagement with GenAI occupied an intermediate position, and Research with GenAI was significantly lower than all three. One plausible interpretation of this pattern is that self-efficacy was strongest where GenAI use sits closest to visible, routine teaching work. Teaching-facing uses, such as designing learning activities or preparing assessments, are more visible in everyday professional life and may give teachers more direct opportunities to develop a stronger sense of capability. Research-facing uses, by contrast, call on different methodological judgements and may be encountered less often in day-to-day teaching. The lower self-efficacy reported for this domain may therefore reflect less familiarity and fewer opportunities for rehearsal, greater task complexity, or some combination of these factors. These explanations were not directly tested in the present study. This interpretation is consistent with the wider language-education literature: Li et al. (2025) found that empirical work on GenAI in language teaching has concentrated on teaching-facing applications, with writing alone accounting for the largest share of studies and research-facing uses receiving comparatively little attention. It also reflects the rationale behind the instrument itself, as Xia et al. (2025) noted that existing competency frameworks tend to overlook the research and assessment dimensions of academic work, which their scale was designed to capture.

The position of the research domain deserves closer attention, because it is here that the study’s sharpest domain-level finding lies. Research with GenAI recorded not only the lowest domain mean (*M* = 2.84) but also a restricted upper range relative to the other domains, with its highest observed value reaching only 4.13 compared with 5.00 in the other three domains. This could indicate that relatively few respondents felt highly capable in research-related uses of GenAI, and the weakness was sharpest in the data-analysis dimension (*M* = 2.73), the lowest of all nine. A plausible reading is that applying GenAI to research design and, especially, to data analysis draws on specialised methodological competencies that are less supported by the kinds of everyday GenAI use through which teaching-facing confidence appears to develop. Zhou et al. (2026), in their review of teacher AI competence, similarly argue that such competence develops unevenly across the dimensions of academic work and that building it requires sustained professional learning rather than incidental exposure alone. This domain-level pattern is important because the overall score does not reveal it.

### 5.4. Predictors of Overall GenAI Competency Self-Efficacy

The third research question asked which background and AI-experience factors were associated with overall GenAI competency self-efficacy. The hierarchical regression returned a clear answer: once prior AI training and frequency of GenAI use were entered, the model accounted for roughly two-thirds of the variance in self-efficacy, whereas the career-background variables entered first explained almost none of it. The two AI-specific variables were the meaningful predictors, with frequency of GenAI use (*β* = 0.533) and prior AI training (*β* = 0.410) each making a strong independent contribution. This pattern fits Bandura’s (1977, 1997) account of self-efficacy as built through mastery experiences: teachers who use GenAI more often and have undergone training may have had more opportunities to attempt GenAI-related tasks, judge the results, and develop a sense of capability. It is also consistent with Hazzan-Bishara et al. (2025), who described a reinforcing cycle in which confidence encourages experimentation with AI tools and that experimentation, in turn, consolidates confidence. The finding also converges with Darko et al. (2026), whose regression on teachers’ GenAI attitudes found that AI-specific confidence carried more weight than general background characteristics.

The relative weight of the two AI-specific predictors warrants a note of caution, however. Frequency of GenAI use emerged as the strongest predictor, but use and self-efficacy are conceptually close: teachers who feel capable may use GenAI more, and frequent users may feel more capable. The association is therefore consistent with a reciprocal relationship rather than evidence that use alone produces confidence. Prior AI training, by contrast, represents a more readily actionable area for institutional support, even though its coefficient was marginally smaller. The career-background variables were not significant once AI-specific experience was included. Teaching experience therefore provided no evidence of a seniority advantage; its slightly negative coefficient should be read only as the absence of a positive seniority effect, not as evidence that experience reduces GenAI self-efficacy. The regression analysis concerned GenAI competency self-efficacy at the overall level only; because the outcome was the GAICS-TR composite score, it does not establish whether the same associations hold within each of the four domains. The broader implication is that GenAI competency self-efficacy in this sample appears tied more to what teachers have done with GenAI than to how long they have been in the profession, echoing Zhou et al.’s (2026) argument that teacher AI competence develops through sustained professional learning rather than accruing automatically with general career experience.

### 5.5. Implications

The clearest implication of these findings is that support for GenAI use is likely to be most effective when it is targeted by domain rather than offered as general familiarisation. Confidence was lowest for research-facing uses, particularly for applying GenAI to data analysis. Professional development that stops at introducing teachers to tools such as ChatGPT is therefore unlikely to address the area where confidence was weakest. A more responsive approach would extend beyond general orientation to the research-facing areas in which the present sample reported the lowest confidence, including the use of GenAI in research design, the interpretation and checking of GenAI-assisted data analysis, and the methodological judgement needed to use such outputs responsibly. This reading is consistent with Zhou et al. (2026), who argued that teacher AI competence develops unevenly and that building it calls for sustained, deliberate professional learning rather than brief or generic exposure.

A second implication concerns who such support is designed for. Because prior AI training and frequency of GenAI use were associated with higher self-efficacy while teaching experience and academic rank were not, there is little basis for assuming that senior staff will report higher GenAI self-efficacy simply because they have spent more time in the profession, or that training is mainly a need of early-career teachers. Support is more defensibly offered across all career stages, reaching experienced and junior teachers alike. The pattern also suggests that structured training is a more institutionally actionable route than encouraging greater use on its own: while frequency of use was the stronger statistical predictor, use and self-efficacy are conceptually close, whereas training is a clearly defined provision that institutions can design and deliver. Recent work in the Saudi context similarly points to the value of structured training, with Alkhateeb et al. (2025) documenting how EFL teachers at two Saudi public universities drew on AI tools for examination preparation while identifying gaps in their readiness to use them well.

More broadly, institutions and researchers assessing teachers’ GenAI competency self-efficacy should consider domain-level composite scores alongside the overall score. This would make areas of lower self-efficacy, such as Research with GenAI, easier to identify.

### 5.6. Limitations and Future Research

Several boundaries should be noted in interpreting these findings. The first concerns sampling and context. Participants were recruited through convenience and snowball sampling within Saudi public universities, and all were EFL teachers, so the sample reflects a particular national, institutional, and disciplinary setting rather than EFL teachers at Saudi public universities as a whole or university teachers more broadly. The survey link was forwarded through professional networks rather than issued from a fixed list, so the response rate could not be determined. Teachers with greater interest in or familiarity with GenAI may also have been more likely to respond, which could bias the self-efficacy estimates upward. Because institutional affiliation was recorded using an optional item and was therefore incomplete, possible within-university dependence could not be fully examined. These features limit the representativeness and generalisability of the findings and should be considered when interpreting the observed estimates. The second concerns the design. The study was designed to provide a cross-sectional account rather than to test causal effects, so the associations of prior AI training and GenAI use frequency with self-efficacy should not be interpreted as evidence that either factor raises self-efficacy. This caution is especially important for use frequency, given its conceptual proximity to self-efficacy and the inability of the design to establish temporal direction. Because the predictors and outcome were self-reported in the same survey at one time point, some associations may be inflated by common-source effects. The Harman diagnostic, which was limited to the 35 GAICS-TR items, cannot rule out common-source inflation in the associations between the predictors and outcome. The AI-training variable also combined training type and amount and should therefore be interpreted as an ordered exposure proxy rather than a measure of training intensity alone. The third concerns what the instrument captures. Because the measure is one of self-efficacy, the findings describe teachers’ perceived capability rather than their demonstrated competence, and a teacher’s confidence in a given area need not coincide with performance in it. The fourth concerns the measurement structure of the adapted GAICS-TR, which remains provisional. Item-level CFA did not provide acceptable evidence for a clean latent structure in this sample. The dimension-level CFA provided supportive evidence for the four-domain scoring structure used in the analyses, but the substantial overlap between Basic GenAI Understanding and Teaching with GenAI limits claims that all four domains are sharply distinct. Accordingly, the domain comparisons should be interpreted as comparisons among pre-specified composite scores rather than as evidence of four fully distinct latent constructs. Further research should examine the item-level structure of the adapted instrument, refine items where necessary, and test measurement invariance in larger and independent samples. These measurement limitations narrow the claims that can be made from the domain-level findings and reinforce the need for further validation.

Future research could build on these findings in several directions. Longitudinal and intervention designs would allow the direction of the training and use relationships to be examined directly, testing whether structured training precedes and supports gains in self-efficacy rather than merely accompanying them. Pairing self-efficacy measures with performance-based or observational evidence would help establish how closely perceived capability tracks demonstrated competence, particularly in the research-facing areas where confidence was lowest. Extending the work to other national, institutional, and disciplinary contexts would clarify how far the domain pattern found here recurs elsewhere. Future research could also examine the factor structure and measurement invariance of the adapted scale across Saudi and wider EFL teacher populations, which would strengthen the basis for comparative work using this instrument.

## 6. Conclusions

This study offered a domain-differentiated account of GenAI competency self-efficacy among EFL teachers at Saudi public universities, using a contextually adapted version of the GAICS-TR. The overall score was just above the scale midpoint, with teachers reporting greater confidence in basic understanding and teaching-related uses than in research-related uses, particularly data analysis. In the main regression, only the two AI-specific variables, namely prior AI training and frequency of GenAI use, were significantly associated with overall self-efficacy. These findings identify a possible professional-development priority: targeted support for research-related applications, made available across career stages as one response to the lower self-efficacy reported in those areas.

## Figures and Tables

**Table 1:** Demographic and professional characteristics of the sample (*N* = 232).

Variable	Category	*n*	%
Gender	Male	123	53.0
	Female	109	47.0
Age group	Under 30	7	3.0
	30–39	108	46.6
	40–49	84	36.2
	50–59	33	14.2
	60 or older	0	0.0
Highest qualification	Bachelor’s degree	36	15.5
	Master’s degree	100	43.1
	PhD/Doctorate	96	41.4
Academic rank/position	Teaching Assistant/Language Instructor	36	15.5
	Lecturer	100	43.1
	Assistant Professor	47	20.3
	Associate Professor	27	11.6
	Professor	22	9.5
University-level teaching experience	Less than 1 year	1	0.4
	1–5 years	12	5.2
	6–10 years	86	37.1
	11–15 years	77	33.2
	More than 15 years	56	24.1

**Table 2:** AI training, GenAI use frequency, and tools selected (*N* = 232).

Variable	Category	*n*	%
AI training	No, none	100	43.1
	Informal/self-directed learning only	65	28.0
	One formal training session or workshop	39	16.8
	Multiple formal training sessions or structured course	28	12.1
Frequency of GenAI use	Never	36	15.5
	Rarely	46	19.8
	Occasionally	56	24.1
	Frequently	56	24.1
	Daily or almost daily	38	16.4
Tool selected	ChatGPT	155	66.8
	Microsoft Copilot	11	4.7
	Google Gemini	54	23.3
	Claude	35	15.1
	DeepSeek	0	0.0
	Grammarly/AI writing assistants	44	19.0

*Note.* Tool categories were not mutually exclusive. Percentages are based on the total sample size.

**Table 3:** Internal consistency of the adapted GAICS-TR.

Scale/Domain	Number of Items	Valid *N*	Cronbach’s *α*
Total GAICS-TR	35	232	0.945
Basic GenAI Understanding	7	232	0.863
Teaching with GenAI	12	232	0.874
Research with GenAI	8	232	0.778
Professional Engagement with GenAI	8	232	0.854

**Table 4:** Confirmatory factor analysis model fit for the adapted GAICS-TR.

Model	Level	Estimator	*χ*^2^(df)	CFI	TLI	RMSEA [90% CI]	SRMR
Four-domain	Item	WLSMV	4270.635 (554)	0.684	0.660	0.170 [0.166, 0.175]	0.164
One-factor	Item	WLSMV	5019.453 (560)	0.620	0.597	0.186 [0.181, 0.190]	0.170
Four-domain	Item	MLR	2132.399 (554)	0.510	0.474	0.111 [0.107, 0.115]	0.189
Four-domain	Dimension	MLR	37.515 (21)	0.989	0.982	0.066 [0.029, 0.099]	0.024
One-factor	Dimension	MLR	229.272 (27)	0.860	0.813	0.180 [0.161, 0.199]	0.079

*Note.* WLSMV = weighted least squares mean- and variance-adjusted; MLR = robust maximum likelihood; CFI = comparative fit index; TLI = Tucker–Lewis index; RMSEA = root mean square error of approximation; SRMR = standardised root mean square residual. *χ*^2^ values are scaled test statistics. For the dimension-level four-domain model, robust MLR CFI, TLI, and RMSEA are reported; SRMR is reported in its standard form. The item-level four-domain MLR model was estimated as an estimator-sensitivity check. A nine-factor item-level model was also estimated but was inadmissible and is described in the text rather than interpreted as fit evidence. All *χ*^2^ tests were significant at *p* < 0.001 except the dimension-level four-domain model, *p* = 0.015.

**Table 5:** Descriptive statistics for overall and domain-level GenAI competency self-efficacy.

Scale/Domain	*N*	*M*	*SD*	95% CI	Min	Max
Overall GAICS-TR	232	3.23	0.64	[3.15, 3.32]	1.23	4.71
Basic GenAI Understanding	232	3.42	0.80	[3.31, 3.52]	1.00	5.00
Teaching with GenAI	232	3.37	0.77	[3.28, 3.47]	1.00	5.00
Research with GenAI	232	2.84	0.62	[2.76, 2.92]	1.00	4.13
Professional Engagement with GenAI	232	3.27	0.73	[3.17, 3.36]	1.00	5.00

**Table 6:** Descriptive statistics for the nine GAICS-TR dimensions.

Domain	Dimension	*N*	*M*	*SD*	95% CI
Basic GenAI Understanding	GenAI Knowledge	232	3.34	0.82	[3.24, 3.45]
Basic GenAI Understanding	GenAI Ethics	232	3.52	0.91	[3.40, 3.64]
Teaching with GenAI	Student Interaction	232	3.29	0.81	[3.19, 3.40]
Teaching with GenAI	Learning Design	232	3.41	0.81	[3.30, 3.51]
Teaching with GenAI	Assessment	232	3.43	0.79	[3.32, 3.53]
Research with GenAI	Research Design	232	2.94	0.66	[2.86, 3.03]
Research with GenAI	Data Analysis	232	2.73	0.67	[2.64, 2.82]
Professional Engagement with GenAI	Professional Development	232	3.25	0.73	[3.16, 3.35]
Professional Engagement with GenAI	Staying Current	232	3.28	0.79	[3.18, 3.38]

**Table 7:** Bonferroni-adjusted pairwise comparisons among the four domains.

Comparison	Mean Difference	*SE*	*p*	95% CI
Basic GenAI Understanding—Teaching with GenAI	0.04	0.03	0.705	[−0.03, 0.12]
Basic GenAI Understanding—Research with GenAI	0.58	0.05	<0.001	[0.45, 0.71]
Basic GenAI Understanding—Professional Engagement with GenAI	0.15	0.04	0.001	[0.05, 0.26]
Teaching with GenAI—Research with GenAI	0.54	0.04	<0.001	[0.42, 0.65]
Teaching with GenAI—Professional Engagement with GenAI	0.11	0.03	0.010	[0.02, 0.20]
Professional Engagement with GenAI—Research with GenAI	0.43	0.04	<0.001	[0.32, 0.54]

*Note. p* values and confidence intervals are Bonferroni-adjusted. Positive values indicate that the first domain in the comparison had the higher mean.

**Table 8:** Correlations between overall GAICS-TR scores and RQ3 predictors.

Predictor	Pearson *r*	*p*	Spearman *ρ*	*p*
University-level teaching experience	−0.077	0.241	−0.058	0.377
Academic rank/position	−0.110	0.096	−0.082	0.214
AI training	0.671	<0.001	0.663	<0.001
Frequency of GenAI use	0.730	<0.001	0.756	<0.001

**Table 9:** Hierarchical regression predicting overall GenAI competency self-efficacy.

Predictor	*B*	*SE B*	*β*	*t*	*p*	95% CI for *B*	VIF
Step 1							
University-level teaching experience	−0.024	0.053	−0.034	−0.454	0.650	[−0.129, 0.081]	1.275
Academic rank/position	−0.052	0.041	−0.094	−1.266	0.207	[−0.132, 0.029]	1.275
Step 2							
University-level teaching experience	−0.058	0.031	−0.081	−1.855	0.065	[−0.119, 0.004]	1.284
Academic rank/position	0.028	0.024	0.051	1.157	0.248	[−0.020, 0.075]	1.311
AI training	0.252	0.028	0.410	9.177	<0.001	[0.198, 0.307]	1.356
Frequency of GenAI use	0.261	0.022	0.533	12.023	<0.001	[0.218, 0.303]	1.334

*Note.* Step 1: *R*^2^ = 0.013, adjusted *R*^2^ = 0.004, *F*(2, 229) = 1.49, *p* = 0.227. Step 2: *R*^2^ = 0.665, adjusted *R*^2^ = 0.659, *F*(4, 227) = 112.80, *p* < 0.001. Change from Step 1 to Step 2: Δ*R*^2^ = 0.652, *F* change (2, 227) = 221.23, *p* < 0.001.

## Data Availability

The dataset generated and analysed during the current study is not publicly available due to participant confidentiality but is available from the corresponding author on reasonable request where ethically permissible.
